# Investigating How Parental Instructions and Protective Responses Mediate the Relationship Between Parental Psychological Flexibility and Pain-Related Behavior in Adolescents With Chronic Pain: A Daily Diary Study

**DOI:** 10.3389/fpsyg.2019.02350

**Published:** 2019-10-17

**Authors:** Melanie Beeckman, Laura E. Simons, Sean Hughes, Tom Loeys, Liesbet Goubert

**Affiliations:** ^1^Department of Experimental Clinical and Health Psychology, Ghent University, Ghent, Belgium; ^2^Department of Anesthesiology, Perioperative and Pain Medicine, Stanford University School of Medicine, Palo Alto, CA, United States; ^3^Department of Data Analysis, Ghent University, Ghent, Belgium

**Keywords:** parental psychological flexibility, adolescent chronic pain, adolescent pain-related behavior, parental protective behavior, parental instructions

## Abstract

**Background:**

Parental behavior can influence how well adolescents cope with chronic pain. Previous research has largely focused on how parents negatively impact adolescent functioning. Yet more recent work suggests that parents – and particularly parental psychological flexibility – can foster better adolescent pain-related functioning. In this study we examined if parental protective responses and instructions to engage in activities in the presence of pain mediate the impact of parental psychological flexibility and acceptance of adolescent pain on adolescents’ daily pain-related behavior.

**Method:**

Fifty-six adolescents with chronic pain (*M*_age_ = 14.5 years, 86% girls) and one of their parents (93% mothers) were recruited at initial evaluation at two pediatric pain clinics in the US. Parents completed baseline questionnaires assessing psychologically flexible parenting and acceptance of adolescent pain. Next, parents and adolescents completed a 14-day self-report diary assessing adolescent activity-avoidance and activity-engagement in the presence of pain (adolescent report), and parental protective responses and instructions for their adolescent to engage in activities (parent report).

**Results:**

Psychologically flexible parenting and acceptance of adolescent pain in parents were indirectly related to lower daily adolescent activity-avoidance, via their negative association with daily parental protective responses. Positive associations also emerged between baseline psychologically flexible parenting and overall levels of adolescent activity-engagement via its negative association with overall levels of parental protectiveness across the 14-day period. Psychologically flexible parenting and parental acceptance of adolescent pain were also indirectly related to daily decreases in adolescent activity-avoidance via their association with daily increases in parental activity-engagement instructions. These baseline parental resilience factors were also positively related to overall levels of parental engagement instructions, a route via which an indirect association with both higher overall activity-engagement as well as higher overall activity-avoidance in the adolescent was observed.

**Conclusion:**

Our findings suggest an (indirect) adaptive role of parental psychological flexibility on adolescent daily pain-related behavior via its impact on parental protective behavior. If our findings replicate, they would suggest that these parental behaviors could be targeted in pain treatments that include both adolescents and their parents. Future research could further examine the impact of parental instructions on pain-related behavior in adolescents with chronic pain.

## Introduction

Approximately one in five children and adolescents experience chronic pain (King et al., 2011), which frequently affects their physical, emotional, and social functioning (Palermo, 2000; Hunfeld et al., 2001; Palermo and Eccleston, 2009). Growing evidence suggests that parents may inadvertently and adversely impact their adolescent’s functioning in the presence of that pain (Logan et al., 2012; Simons et al., 2015; Chow et al., 2016). Two lines of thought have emerged to explain how parents exert such a negative influence. The first argues that parents tend to emit protective behaviors (e.g., keeping the child home from school) when faced with their adolescent in pain, and that these behaviors directly result in heightened adolescent avoidance of pain-related activities (Palermo and Chambers, 2005; Goubert and Simons, 2013). It is this heightened and persistent avoidance which is assumed to increase risk of disability (Asmundson et al., 2012; Simons and Kaczynski, 2012). A second line of thought argues that adolescent behavior is indirectly influenced by how parents think, act, and feel. For instance, parental fear and catastrophizing about adolescent pain can indirectly influence how much their adolescent avoids pain-related activities through their impact on both parent (i.e., parent pain avoidance) and adolescent psychosocial responses to pain (e.g., adolescent pain-related fear and catastrophizing) (Vowles et al., 2010; Simons et al., 2015). Observational learning processes have been proposed to explain these indirect influences from parent to adolescent functioning (see Goubert et al., 2011; Goubert and Simons, 2013). However, *instructional learning processes* may be an alternative route through which parents may exert an indirect influence upon their adolescent’s functioning. Verbal information – namely – the rich variety of pain-related instructions and rules communicated from parent to adolescent can exert a powerful influence on adolescents’ pain-related behavior even in the absence of direct pain experiences (for more details, see Beeckman et al., 2019a). Parents are uniquely positioned to provide frequent verbal information regarding the potential positive or aversive outcomes of engaging in, or avoiding pain-related activities. Although theoretical work on the relationship between verbal processes and pain has started to emerge (e.g., Bennett et al., 2015; Maeda et al., 2018; Beeckman et al., 2019a), no empirical work has examined if parental verbal instructions guide adolescent pain-related behavior, and ultimately, their functioning over time.

As we mentioned above, most work on the role of parents in the context of pediatric chronic pain has focused on their *maladaptive* influence. Yet parents might also foster resilient functioning in adolescents (i.e., “effective functioning despite stressful circumstances [such as chronic pain]”; Karoly and Ruehlman, 2006; Sturgeon and Zautra, 2010; Goubert and Trompetter, 2017). Admittedly, research supporting this idea is sparse. But several recent studies have started to identify specific parental factors that may increase adaptive functioning in adolescents with chronic pain (e.g., adaptive parenting; see also Cousins et al., 2015; Goubert and Trompetter, 2017; Feinstein et al., 2018; Ross et al., 2018). Parental psychological flexibility is thought to represent one such factor (e.g., McCracken and Gauntlett-Gilbert, 2011; Smith et al., 2015; Wallace et al., 2015; Timmers et al., 2019). Psychological flexibility refers to “being aware of, and open to unwanted and uncontrollable experiences (e.g., seeing your child suffering with chronic pain), while still having the ability to act in line with broader life values (e.g., being an encouraging parent)” (Hayes et al., 2006; McCracken and Gauntlett-Gilbert, 2011; McCracken and Morley, 2014; Vowles et al., 2014). Parents can show psychological flexibility in how they parent in general, or more specifically, for instance, in how they navigate thoughts and feelings that emerge when confronted with their adolescent’s pain. Parental acceptance of adolescent pain is a sub-component of psychological flexibility and the one that has garnered the greatest attention in the pediatric pain literature (e.g., Smith et al., 2015; Feinstein et al., 2018). In contrast, the six processes that constitute psychological flexibility in parents (i.e., *acceptance, defusion, being present, self-as-context, values-based action, committed action;* see also McCracken and Morley, 2014) and the role of psychological flexibility in parent-child interactions in particular (e.g., Greene et al., 2015; Wallace et al., 2015; Brassell et al., 2016) have received far less attention. Several studies indicate that parental psychological flexibility in the context of adolescent chronic pain is associated with lower levels of adolescent disability and depression (McCracken and Gauntlett-Gilbert, 2011; Smith et al., 2015; Wallace et al., 2015). Such work suggests that the relationship between parental psychological flexibility and adolescent functioning may itself be mediated by lower parental protective responses and higher adolescent acceptance of pain (Smith et al., 2015; Timmers et al., 2019). We build on this prior work and introduce another possibility: the verbal information that parents communicate to their adolescent (e.g., “It is important that you engage in activities that you value even though you are pain”) may represent yet another way via which parental psychological flexibility exerts an influence on adolescent functioning.

With this in mind, the current study examined - using a diary design – the relations between parents’ psychological flexibility in the interactions with their adolescent in general and acceptance of adolescent pain, and daily activity-avoidance and activity-engagement in adolescents with chronic pain. First, we expected that higher psychologically flexible parenting and higher parental acceptance of adolescent pain would be indirectly related to lower daily adolescent activity-avoidance via lower parental protective responses displayed on a daily basis. Likewise, it was explored if psychological flexible parenting and parental acceptance of adolescent pain would be indirectly related to higher daily adolescent activity-engagement via lower parental protective responses. Second, we explored if the type of parental verbal instructions directed at their adolescent also mediated these relationships. Our exploratory hypothesis was that higher levels of parental instructions to engage in pain-related activities would mediate the relationship between parental psychological flexibility and parental acceptance of adolescent pain on the one hand and daily adolescent behavior (i.e., lower avoidance and higher activity engagement) on the other hand. Examining the processes that underlie the influence of parental psychological flexibility on adolescent functioning on a daily basis may help to advance our understanding of its adaptive effects and help to identify (novel) targets for treatments directed at enhancing adolescent and parent functioning in the context of chronic pain in youth.

## Materials and Methods

### Participants

Participants were adolescents with mixed chronic pain conditions and their primary caregiver (i.e., mother or father). Recruitment took place when they presented for initial clinical evaluation at the Pain Treatment Service at Boston Children’s Hospital (BCH) between February 2017 and December 2017, and via the Pediatric Pain Management Clinic at Stanford Children’s Health (SCH) between February 2017 and February 2018. Institutional Review Board (IRB) approval was granted at each site prior to the start of the study (BCH IRB#P0020989; Stanford IRB#39092). The present study is part of a large research project, Child Pain In Context (CP-IC), with the complete study protocol available at http://hdl.handle.net/1854/LU-8578159. One paper has been published already on this CP-IC research project and examined adolescent predictors of pain-related behavior using a network analysis approach (see Beeckman et al., 2019b). The current paper focuses on parental variables impacting adolescent pain-related behavior.

Eligibility criteria for participation were [1] being 11–17 years old, [2] reporting persistent or recurrent pain for 3 months or longer, [3] having internet access at home or on an accessible smartphone, [4] absence of significant cognitive impairments (e.g., intellectual disability, severe brain injury), [5] absence of severe psychiatric or neurological conditions, and [6] availability of one primary caregiver who was also willing to participate.

Of the 84 parent-adolescent dyads who initially consented/assented to participate, 56 dyads (i.e., 67%) completed a set of baseline self-report questionnaires followed by a 14-day diary assessment period. Reasons for non-completion included: lack of interest after initial consent (*n* = 19) and no baseline parent data (*n* = 9). As specified in the CP-IC protocol, at least 50 participants (i.e., parent-adolescent dyads) should be sufficient to perform multilevel analyses (Maas and Hox, 2005; Nezlek, 2012).

### Study Procedure

Informed consent was obtained on paper or online before the start of the study. Parents signed an informed consent for their own participation and that of the adolescent, and adolescents additionally gave informed assent. All study data were collected and managed using the REDCap (Research Electronic Data Capture) (Harris et al., 2009) tool hosted at BCH and Stanford University. REDCap is a secure, web-based application designed to support data capture for research studies. All communication with the participants was carried out via the parent (either via text message or e-mail).

At the start of the study participants received an online link to access the baseline self-report questionnaires. Once self-reports were completed, the diary period was scheduled to begin the following week. Automatic messages containing the diary surveys were sent to the participants each day for 14 consecutive days. Adolescents were asked to complete surveys in the afternoon and the evening, while parents completed one end-of-the-day diary. Afternoon surveys for the adolescent were sent at 2 pm and deactivated at 6 pm, and evening surveys for adolescent and parent were sent at 6 pm and deactivated at 10 am the next day. In line with the recommendation by Nezlek (2012, p. 46), all surveys completed between these time windows were treated as valid reports. If an adolescent and/or parent did not complete any of the required diary assessments on three consecutive days (despite reminder calls), the family was given the option of withdrawing from the study. If they decided to continue and the adolescent and/or parent failed to provide data on any additional days after this final reminder, their participation in the study was terminated and they received no further diary invitations. It was not possible for adolescent and/or parent to continue their participation individually.

Participants who started the 2-week diary period received one 10-dollar gift voucher (1 per family) at the end of the first week irrespective of the number of completed days. This was intended to serve both as a sign of appreciation for their participation, as well as an incentive to complete daily diaries in the second week. Participating parent-adolescent pairs received a 20-dollar gift voucher at the end of week two unless they withdrew from the study during the first week.

### Measures

#### Baseline Questionnaires

Adolescents and their parents completed a set of self-report questionnaires measuring demographic information and key study variables prior to the start of the diary period.

*Demographic information* was obtained by asking adolescents and parents to complete a short questionnaire assessing adolescent age, gender, ethnicity, race, and schooling grade. Parents were additionally asked to report on adolescent pain characteristics (i.e., pain location and duration) and parent gender, marital status, and educational level.

*Adolescent pain severity* was assessed by means of the child version of the Graded Chronic Pain Scale (Von Korff et al., 1992; Vervoort et al., 2014). Current and average pain intensity in the past six months were rated on a 11-point numerical rating scale (0 = *no pain*, 10 = *worst possible pain*) and used to calculate a characteristic pain intensity score. Disability was measured in terms of disability points. These points reflect a sum score of points allocated to the total number of days on which the child was prevented from carrying out usual activities in the past six months (0: <7 days; 1: ≥7 and <15 days; 2: ≥15 and <31 days; 3: ≥31 days) and points allocated to the degree to which pain caused difficulties in performing their usual activities in that same period (0 = *no difficulties at all*; 10 = *impossible to do activities*; 0: <3; 1: ≥3 and <5; 2: ≥5 and <7; 3: =≥ 7). Based on the scores for pain intensity and disability adolescents can be classified into 5 pain grades (0 = pain free; I = low disability [<3], low intensity [<5]; II = low disability [<3], high intensity [≥5]; III = moderate disability [3 or 4], regardless of pain intensity; IV = high disability [≥5] regardless of pain intensity) which was used to describe the sample (Vervoort et al., 2014). The GCPS has been used as a valid measure of pain severity in primary care, chronic pain, and general population samples (Von Korff et al., 1993; Smith et al., 1997; Elliott et al., 2000). The child version has shown good psychometric properties in a general population sample (Vervoort et al., 2014).

*Psychologically flexible parenting* was measured by the Parental Acceptance Questionnaire (6-PAQ; Greene et al., 2015). The 6-PAQ was developed to measure the six core processes that constitute psychological flexibility applied to an interpersonal, parenting context. The questionnaire consists of 18-items that are answered on a 4-point response scale (1 = *strongly disagree/never*; 4 = *strongly agree/almost always*). A total score and subscale scores for each of the six processes can be obtained. Example items for each of the six subscales are: “It is difficult to initiate/maintain routines because I don’t want to deal with my child’s reactions” (*Acceptance*); “I have negative thoughts about myself when my child behaves in a negative way” (*Defusion*); “I feel like my mind is somewhere else when I play with my child” (*Being Present*); “When parenting doesn’t go as I had planned, I feel like a failure” (*Self-as-Context*); “My actions as a parent are consistent with my values” (*Value-based Actions*); and “My parenting behaviors are based on what matters to me as a parent rather than how I feel in the moment” (*Committed Action*). In line with previous research (see Williams et al., 2012; Beeckman et al., 2018), items were reverse-scored so that higher total scores reflect higher psychologically flexible parenting. The 6-PAQ has been shown to be a psychometrically sound measure to assess psychological flexibility in the parenting of young, healthy children (3–12 years) (Greene et al., 2015). To the best of our knowledge, this was the first study to use the 6-PAQ to assess parenting-specific psychological flexibility in parents of adolescents (11–17 years) with chronic pain. Cronbach’s alpha for the total 6-PAQ scale in the current study was 0.83.

*Parental acceptance of adolescent pain* was assessed by means of the Parent Pain Acceptance Questionnaire (PPAQ; Smith et al., 2015). The PPAQ consists of two subscales measuring a parent’s acceptance of pain-related thoughts and feelings [four items; e.g., “I must change my thoughts and feelings about my child’s pain before I can take important steps in my life (reverse scored)”], and a parent’s activity-engagement despite their adolescent’s pain (11 items; e.g., “I lead a full life even though my child has chronic pain”). All items were scored on a 5-point response scale (0 = *never true*; 4 = *always true*). Higher total scores reflect higher parent acceptance of child pain. The PPAQ has been validated in a sample of parents of children with chronic pain (Smith et al., 2015). In the current study Cronbach’s alpha was 0.91 for the total PPAQ scale.

#### Daily Diary Measures

Daily adolescent pain intensity, activity-avoidance and activity-engagement, and parental protective behavior and engagement instructions were measured by means of 14-day daily diary for adolescents and parents. Adolescents were asked to report on “the period since the previous diary entry” in the afternoon and evening assessments. Parents were asked to report on their experiences “today” in their daily diaries. All diary items were rated on a five-point response scale (unless stated otherwise) with the following labels: 0 (*not at all true*), 1 (*a little true*), 2 (*somewhat true*), 3 (*mostly true*), and 4 (*totally true*). Diary items were developed by the research team based on items of existing questionnaires that were adjusted for daily or momentary use and consequently validated using the Discriminant Content Validity (DCV) procedure of Johnston et al. (2014). As a part of this content validation procedure five psychologists with expertise in the field of pediatric pain research were asked to rate the extent to which each of the diary items measured the predetermined constructs to illuminate those that required reformulation before inclusion in the final diary. None of the items that were developed to measure the constructs used in this study required reformulation based on the results of the expert ratings. Total diary scale scores were calculated by taking the average of the single item responses (i.e., if the scale consisted of two or three items), but only if at least 75% of the items were completed. If less than 75% of the items were completed, the total scale score was not calculated and considered missing. To effectively answer the key research questions, a daily score was calculated for each adolescent variable by taking the average of the afternoon and evening scale scores.

##### Daily adolescent pain intensity

Adolescents were asked about their overall level of pain in the afternoon and evening with 1 item (“Since the previous diary entry, what was your overall level of pain?”). This item was rated on a 11-point numerical rating scale from 0 (*no pain*) to 10 (*worst possible pain*), which is considered to be the golden standard for the assessment of pain intensity (Castarlenas et al., 2017).

##### Daily adolescent activity-avoidance

Adolescents’ avoidance of activities because of the pain in the afternoon and evening was assessed using three items that were based on the ‘Avoidance of Activities’ subscale of the Fear of Pain Questionnaire for Children [FOPQ-C (Simons et al., 2011)] and adjusted for use in the diary (“I skipped my planned activities because I expected them to trigger or increase my pain.”, “I stopped what I was doing because my pain started to get worse,” “I spent my time resting instead of doing my activities, because of my pain”). These items were selected to reflect different types of pain-related avoidance strategies in agreement with the author of the original FOPQ-C, and were evaluated as valid items by the experts during the content validation procedure. Good internal consistency (α = 0.86) and reliability have been found for the FOPQ-C avoidance subscale in pediatric chronic pain samples (Simons et al., 2011).

##### Daily adolescent activity-engagement

Adolescents were asked to complete two items that assessed their engagement in activities in the presence of pain in the afternoon and evening. The items of the activity-engagement scale were only presented to those who experienced some level of pain at the same time (i.e., a pain intensity score of one or higher). This is in accordance with the operationalization of activity-engagement as a behavior which is only relevant in the presence of pain. Following items were used: “I have put effort into completing activities that I find important or fun, while I was in pain,” and “I persisted in carrying out my planned activities while I was in pain.” These daily items were developed based on items of the ‘Activity-engagement’ subscale of the Chronic Pain Acceptance Questionnaire for Adolescents (CPAQ-A) (McCracken et al., 2010) and were evaluated as valid items by the experts during the content validation procedure. The CPAQ-A has proven to be a valid and reliable measure of pain acceptance (i.e., pain willingness and activity-engagement) in youth with chronic pain (McCracken et al., 2010; Wallace et al., 2011).

##### Daily parental protective responses

Parents reported daily on their protective responses toward the adolescent in pain, by means of two items: “Today, I made sure that my child did not have to do certain activities (e.g., household chores) because of his/her pain” and “Today, I canceled my personal activities (e.g., job-related duties, household chores and/or hobbies) so that I could be with my child.” These daily items were constructed based on items of the ‘Solicitousness’ subscale of the Inventory of Parent/Caregiver Reponses to the Children’s Pain Experience (IRPEDNA) (Huguet et al., 2008) and were evaluated as valid items by the expert team. The IRPEDNA has shown good psychometric properties in a sample of parents of healthy children and adolescents from 6 to 16 years (Huguet et al., 2008).

##### Daily parental engagement instructions

Parents were asked to report on the degree to which they provided their child with instructions to either engage in or avoid activities during the past day. The following items were used to assess this: “I told my child to stop or cancel activities when in pain” (activity-avoidance instruction) and “I told my child to keep doing fun or important activities (and other activities he/she usually does) when in pain” (activity-engagement instruction). These items were constructed by reformulating the items of the activity-engagement and avoidance scales in the adolescent diary to represent possible instructions parents might give to their children in the context of pain. We know of no other existing questionnaire measuring parental instructions in the context of pain. A relative parental activity-engagement instruction score was created by subtracting the daily activity-avoidance instruction score from the daily activity-engagement instruction score. A positive score on a given day indicates that a parent provided more instructions to their adolescent to engage in activities despite the pain than activity-avoidance instructions.

### Data Analytic Strategy

Descriptive statistics, correlations, and internal consistencies of the baseline questionnaires were calculated using SPSS (v.25; IBM Statistics). Reliability of the diary scales was calculated in Mplus following a multilevel confirmatory factor analysis framework which makes it possible to estimate within- and between-level reliabilities of the scales (Geldhof et al., 2014). Pearson product-moment correlations were calculated to examine bivariate associations between adolescent age, adolescent gender, psychologically flexible parenting, parental acceptance of adolescent pain, parent and adolescent diary variables (aggregated over days). These correlations were evaluated at the 5% significance level. Multilevel mediation analyses were performed in R (v. 3.5.2; R Foundation of Statistical Computing) using the *lme4*-package (Bates et al., 2015), and 95% confidence intervals for the indirect effects were obtained using the *boot*-package (Davison and Hinkley, 1997; Canty and Ripley, 2019). Multilevel modeling can account for the hierarchical data structure (i.e., multiple observations nested within dyads) without violating the assumption of independence of observations and assumes that observations are missing at random (Snijders and Bosker, 2012).

**FIGURE 1:**
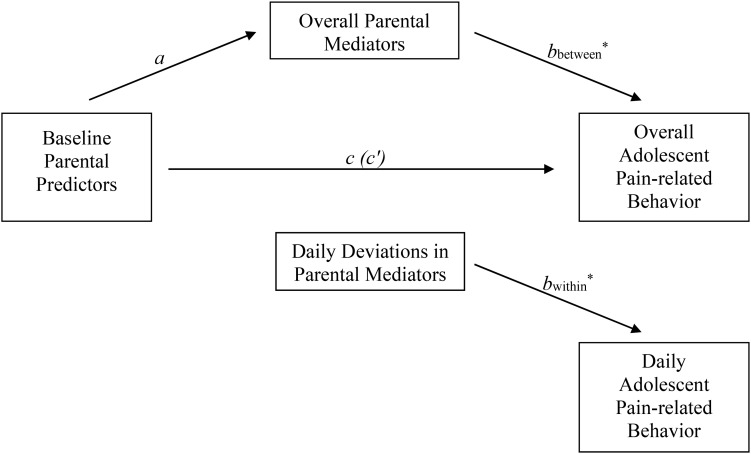
Mediation model structure. This figure shows the general structure of each of the eight mediation models being tested to answer our research questions. Each of these models includes one predictor (Psychologically Flexible Parenting or Parental Acceptance of Adolescent Pain), one mediator (Overall/Daily Parental Protective Responses or Overall/Daily Parental Engagement Instructions) and one outcome (Overall/Daily Adolescent Activity-Avoidance or Overall/Daily Adolescent Activity-Engagement). The representation of within- and between mediation effects is based on how this is done by Zhang et al. (2008). *a*, effect predictor on mediator; *b*, effect mediator on outcome; *c*, total effect predictor on outcome; *c*′, direct effect predictor on outcome (controlled for indirect effect); *a x b*, indirect effect predictor on outcome via mediator.

Figure 1 presents the general structure of each of the mediation models that were fitted to answer our research questions. Predictors (psychologically flexible parenting or parental acceptance of adolescent pain), mediators (parental protective responses or parental instructions concerning activity-engagement), and outcomes (adolescent activity-avoidance or activity-engagement in the presence of pain) were entered separately into the models, resulting in eight mediation models. Adolescent age, gender, and aggregated daily pain intensity scores were explored as potential confounding variables in each model, and were only included as control variables in the final model when they significantly correlated with both the predictor/mediator and the outcome variable. Level 2 predictors (i.e., psychological flexible parenting, parental acceptance of adolescent pain, and adolescent age) were standardized to facilitate interpretation of the coefficients. Random intercepts were allowed, while the slopes of the investigated effects were fixed.

Our longitudinal (daily diary) data allowed us to examine the aforementioned relationships both within- and between parent-adolescent dyads [we adopted this strategy based on a recommendation by Zhang et al. (2008)]. That is, we split the mediator into two independent pieces: the within-dyad deviations and the between-dyad overall means (Figure 1). Within-dyads effects were examined by analyzing the association between daily deviations from the parent-specific average and daily adolescent outcome variables within parent-adolescent dyads. Between-dyads effects were examined by analyzing the association between overall parent mediator and overall adolescent outcome variables (i.e., by taking the average of all daily observations within parents and adolescents across the 14-day diary period). Within-dyads effects reflect that part of the indirect effect of the predictor on the outcome that is explained by the daily variability in the mediator within a given parent-adolescent dyad. In other words, that part of the effect explained by “state” or momentary levels of the mediator variable, i.e., answering questions about when changes occur within the parent-adolescent dyad. Between-dyads effects reflect that part of the indirect effect explained by the variability between parent-adolescent dyads in the “trait” or characteristic level of the mediator variable (for similar terminology see Geiser et al., 2013), i.e., answering questions about how parent-adolescent dyads differ from each other. Whereas cross-sectional data can only address between-dyads effects, the longitudinal nature of our data allowed us to disentangle mediation effects at a within-dyads and between-dyads level. The above-described analyses were focused on examining associations between parent and child variables on a daily basis, but did not examine within-day or day-to-day associations between those variables. Finally, a bootstrap procedure was used to assess the significance of the indirect effects. When weight *a* represents the effect of the predictor on the mediator, and weight *b* the effect of the mediator on the outcome, the indirect effect of the predictor on the outcome (via the mediator) is obtained as the product of weight *a* and *b* (see Figure 1). Significance is determined by inspecting the percentile-based 95% confidence intervals around this product: effects are considered to be significant if this confidence interval does not contain zero.

## Results

### Sample Characteristics

The final sample consisted of 56 adolescent (*M*_age_ = 14.50, *SD* = 1.90) and parent (93% mothers) dyads. The majority of the sample was female (i.e., 86% adolescent girls) and Caucasian (i.e., 66%). Fifty-five percent of adolescents reported musculoskeletal pain (i.e., in the arms, shoulders, neck, or legs) as their primary pain, followed by abdominal pain (i.e., 20%), headaches (i.e., 13%), and other types of pain (i.e., 13%; e.g., pelvic pain). About half of adolescents (i.e., 52%) reported high levels of disability (i.e., pain Grade VI; see section ‘Measures’). Detailed demographic characteristics of adolescents and parents can be found in Table 1.

**TABLE 1:** Sociodemographic characteristics about adolescents and parents.

**Demographic variables**	***M* (*SD*) or % (*N*)**
**Adolescent characteristics**	
Age (years)	14.50 (1.90)
**Gender**	
Female	85.7 (48)
Male	14.3 (8)
**Race**	
White or Caucasian	66.1 (37)
Black or African American	3.6 (2)
Asian	1.8 (1)
Multiracial	3.6 (2)
Choose to not answer	1.8 (1)
Missing	23.1 (13)
**Primary Pain**	
Headache	12.5 (7)
Abdominal Pain	19.6 (11)
Musculoskeletal Pain	55.4 (31)
Other	12.5 (7)
Pain duration (months)	26.59 (23.10)
**Pain grades**	
Grade 0	0 (0)
Grade I	10.7 (6)
Grade II	12.5 (7)
Grade III	21.4 (12)
Grade IV	51.8 (29)
**Parent Characteristics**	
**Relation to child**	
Mother	92.9 (52)
Father	7.1 (4)
**Ethnic background**	
Hispanic	12.5 (7)
Non-Hispanic	85.7 (48)
Missing	1.8 (1)
**Marital status**	
Married	71.4 (40)
Divorced	12.5 (7)
Separated	3.6 (2)
Never Married	12.5 (7)
**Employment status**	
Full-time	51.8 (29)
Part-time	23.2 (13)
Homemaker	17.9 (10)
Unemployed	3.6 (2)
Disabled	3.6 (2)
**Education level**	
High school or less	5.4 (3)
Some college/Vocational school	10.7 (6)
College degree	44.6 (25)
Graduate/Professional school	39.3 (22)

*Grade 0 = pain free; Grade I = low pain intensity, low disability; Grade II = high pain intensity, low disability; Grade III = moderate disability regardless of pain intensity; Grade IV = high disability regardless of pain intensity.*

Of a total of 784 possible daily diary observations (i.e., one observation per day/per participant for 14 consecutive days), 625 data points were available for daily adolescent activity-avoidance (i.e., 20% missing), 528 for daily adolescent activity-engagement (i.e., 32% missing), 582 for parental daily protective responses (i.e., 26% missing), and 560 for parental daily engagement instructions (i.e., 28% missing). Ninety-one percent of the daily pain intensity ratings during the 2-week period were scored at one or higher, while 62% of the daily pain ratings were scored at 4 or higher.

### Descriptive Statistics

Means, standard deviations and bivariate Pearson correlation coefficients between baseline measures of adolescent age, gender, parent variables, and aggregated (adolescent and parent) diary variables can be found in Table 2. Correlational patterns showed a positive association between psychological flexible parenting and parental acceptance of adolescent pain (*r* = 0.38, *p* = 0.004). Adolescent baseline pain intensity and daily activity-avoidance behavior (aggregated over days) were positively correlated (*r* = 0.27, *p* = 0.044). Age, gender, and baseline pain intensity were no significant confounders of any of the investigated relations between parental variables and daily adolescent pain-related behavior and were therefore not included as control variables in the final models.

**TABLE 2:** Sample size, range, means, standard deviations and bivariate pearson correlation coefficients between baseline variables and aggregated daily diary scores.

**Variable**	**N**	**Range**	**M (SD)**	**2**	**3**	**4**	**5**	**6**	**7**	**8**	**9**
**Baseline measures**											
(1) Adolescent Age	56	11 – 17	14.50 (1.90)	0.11	0.05	0.02	0.02	0.11	–0.14	–0.07	–0.04
(2) Adolescent Gender	56	n/a	n/a	–	0.14	0.04	–0.08	–0.01	–0.03	0.16	–0.09
(3) Adolescent Pain Intensity	56	0.3 – 10	5.10 (2.31)	–	–	0.15	–0.19	0.22	–0.04	0.27^∗^	–0.19
(4) Psychologically Flexible Parenting	56	18 – 47	28.67 (6.44)	–	–	–	0.38^∗∗^	–0.15	0.17	–0.10	0.10
(5) Parental Acceptance of Adolescent Pain	56	8 – 56	34 (11.52)	–	–	–	–	–0.44^∗∗^	–0.05	–0.09	0.15
**Diary measures^a^**											
(6) Parent Protective Responses	55	0 – 4	0.52 (0.85)	–	–	–	–	–	–0.14	0.27	–0.10
(7) Parent Engagement Instructions	55	−3.25 – 3.83	1.60 (1.60)	–	–	–	–	–	–	–0.22	0.65^∗∗^
(8) Adolescent Activity-avoidance	56	0 – 4	0.94 (0.86)	–	–	–	–	–	–	–	−0.30^∗^
(9) Adolescent Activity-engagement	56	0.3 – 4	2.70 (1.01)	–	–	–	–	–	–	–	–

*^*a*^Aggregated scores (over days). ^∗^*p* < 0.05, ^∗∗^*p* < 0.01.*

Reliability assessment showed acceptable to excellent within- and between-level reliabilities for the diary scales (see Table 3).

**TABLE 3:** Within- and between-dyads reliabilities for the diary scales.

	**Parent**	**Adolescent**			
	**Protective responses**	**Activity-avoidance**		**Activity-engagement**	
	**Evening**	**Afternoon**	**Evening**	**Afternoon**	**Evening**
Within-dyads α	0.65	0.82	0.81	0.63	0.73
Between-dyads α	0.93	0.95	0.93	0.92	0.93

*Reliabilities are shown for the scales (>2 items) that were used in the diary. Afternoon and evening scores were averaged to obtain a daily activity-avoidance respectively activity-engagement score. Scale reliabilities are calculated based on a procedure by Geldhof et al. (2014).*

### Examining the Indirect Relationship Between Psychologically Flexible Parenting/Parental Acceptance of Adolescent Pain and Adolescent Pain-Related Behavior via Parental Protective Responses

#### Does Psychologically Flexible Parenting Indirectly Impact Daily Adolescent Pain-Related Behavior via Parental Protective Responses?

The left column of Table 4 shows the results of bootstrap analyses designed to test the hypothesized indirect effects from psychologically flexible parenting to daily pain-related behavior in adolescents via parental protective responses. Results showed that there was a significant indirect effect of psychologically flexible parenting on daily adolescent activity-avoidance, via daily parental protective responses, at the within-dyads level (*a* × *b* = −0.03, 95% CI = −0.06 to −0.01), but not at the between-dyads level (*a* × *b* = −0.03, 95% CI = −0.06 to 0.01) (also see Table 4). This suggests that parental psychological flexibility was predictive of lower daily parental protectiveness, and that daily decreases in parental protectiveness *within a parent-adolescent dyad* was associated with decreased levels of adolescent activity-avoidance. Critically, this mediation was not explained by differences *between parents* in their overall level of protectiveness (across the 14-day period), but only by daily variation in protective responding. After controlling for this indirect effect via parental protective responses, results showed no remaining direct effect of psychologically flexible parenting on daily adolescent activity-avoidance (*c*′ = −0.05, 95% CI = −0.12 to 0.05) (Table 4).

**TABLE 4:** Bootstrap tests of indirect effects of psychologically flexible parenting and parental acceptance of adolescent pain on adolescent pain-related behavior via parents’ protective responses.

	**Baseline predictor:**			
	**Psychologically flexible parenting**		**Parental acceptance of adolescent pain**	
**Effect (path)**	**Estimate**	**95% CI**	**Estimate**	**95% CI**
Predictor - > parental protective responses (a-path)	–0.15	−0.20 to −0.09	–0.41	−0.47 to −0.35

**Parental Protective Responses - > Outcome (b-path)**				

**Adolescent activity-avoidance**				
*Within-dyad*	0.20^†^	0.04 to 0.37	0.20^†^	0.03 to 0.37
*Between-dyad*	0.18	−0.04 to 0.33	0.20	−0.05 to 0.41
Adolescent Activity-engagement				
*Within-dyad*	–0.11	−0.25 to 0.02	–0.11	−0.25 to 0.03
*Between-dyad*	−0.20^†^	−0.36 to −0.04	–0.17	−0.37 to 0.06

**Indirect effect via parental protective responses (a^∗^b)**				

**Adolescent activity-avoidance**				
*Within-dyad*	−0.03^†^	−0.06 to −0.01	−0.08^†^	−0.18 to −0.01
*Between-dyad*	–0.03	−0.06 to 0.01	–0.08	−0.17 to 0.02
**Adolescent activity-engagement**				
*Within-dyad*	0.02	−0.00 to 0.04	0.05	−0.01 to 0.10
*Between-dyad*	0.03^†^	0.01 to 0.06	0.07	−0.02 to 0.15
**Total effect predictor → outcome (c-path)**				
Adolescent activity-avoidance	–0.04	−0.11 to 0.04	–0.01	−0.09 to 0.08
Adolescent activity-engagement	0.09	0.03 to 0.15	0.16	0.09 to 0.24
**Direct effect predictor → outcome (c′-path) ^1^**				
Adolescent activity-avoidance	0.02	−0.05 to 0.09	0.06	−0.06 to 0.17
Adolescent activity-engagement	0.00	−0.08 to 0.09	0.06	−0.05 to 0.18

*We refer to the template model in Figure 1 for additional help in interpreting the results presented in this table. ^1^After controlling for indirect effect. Effects indicated with ^†^ were no longer significant when applying a conservative Bonferroni correction for multiple testing.*

Analyses also revealed an indirect effect of psychologically flexible parenting on daily adolescent activity-engagement, via parental protective responses, but only at the between-dyads level (*a* × *b* = −0.03, 95% CI = 0.01 to 0.06) (Table 4). This suggests that the indirect effect was explained by differences between parent-adolescent dyads in the overall level of protectiveness in parents and not by daily variation in parental protective responses within those dyads. There was no significant direct effect of psychologically flexible parenting on daily adolescent activity-engagement after controlling for the indirect effect via parental protective responses (*c*′ = 0.07, 95% CI = −0.01 to 0.14) (Table 4).

#### Does Parental Acceptance of Adolescent Pain Indirectly Impact Daily Adolescent Pain-Related Behavior via Parental Protective Responses?

The hypothesized indirect effect of parental acceptance of adolescent pain on daily adolescent activity-avoidance via parental protectiveness was significant at the within-dyads level (*a* × *b* = −0.08, 95% CI = −0.18 to −0.01), but not at the between-dyads level (*a* × *b* = −0.08, 95% CI = −0.17 to 0.02) (see Table 4, right column). This suggests that the indirect effect was explained by daily variation in parental protective responses within parent-adolescent dyads, but not by differences between parents in terms of their overall protectiveness across the 14-day diary period. After controlling for the indirect effect via parental protective responses, there was no significant direct effect of parental acceptance of adolescent pain on daily adolescent activity-avoidance (*c*′ = 0.06, 95% CI = −0.06 to 0.17) (Table 4).

Finally, no significant indirect effect of parental acceptance of adolescent pain on daily adolescent activity-engagement via parental protective responses was observed (Table 4). The direct effect of parental acceptance of adolescent pain on daily adolescent activity-engagement was also not significant after controlling for daily parental protective behavior (*c*′ = 0.06, 95% CI = −0.05 to 0.18) (Table 4).

### Examining the Indirect Effect of Psychologically Flexible Parenting/Parental Acceptance of Adolescent Pain on Daily Pain-Related Behavior in Adolescents via Parental Instructions

#### Does Psychologically Flexible Parenting Indirectly Impact Daily Adolescent Pain-Related Behavior via Parental Engagement Instructions?

The indirect effect of psychologically flexible parenting on daily adolescent activity-avoidance via parental engagement instructions was significant, both at the within- (*a* × *b* = −0.06, 95% CI = −0.13 to −0.002) and between-dyads level (*a* × *b* = 0.09, 95% CI = 0.003 to 0.16) (see Table 5, left column). Critically, however, the direction of effect was opposite at the within and between-dyads levels. On the one hand, there was a *negative* within-dyads indirect effect of psychologically flexible parenting on daily adolescent activity-avoidance, indicating that psychologically flexible parenting was associated with less daily adolescent activity-avoidance. This was explained by increased levels of daily parental engagement instructions (*b*_within_ = −0.03, 95% CI = −0.06 to −0.001). This suggests that psychologically flexible parenting was predictive of higher daily engagement instructions, and that daily increases in engagement instructions within the parent-adolescent dyad were associated with decreased levels of adolescent activity-avoidance. On the other hand, we also found an unexpected *positive* indirect effect of parental psychological flexibility on adolescent activity-avoidance at the between-dyads level, via higher overall levels of parental engagement instructions (*b*_between_ = 0.04, 95% CI = 0.001 to 0.08). This suggests that psychologically flexible parenting was also predictive of higher overall levels of engagement instructions in parents and that these higher overall levels were associated with higher overall adolescent activity-avoidance across the 14-day period. After controlling for the indirect effects of parental psychological flexibility via daily parental engagement instructions, no significant direct effect of parental psychological flexibility on daily adolescent activity-avoidance emerged (*c*′ = −0.05, 95% CI = −0.13 to 0.04) (Table 5).

**TABLE 5:** Bootstrap tests of indirect effects of psychologically flexible parenting and parental acceptance of adolescent pain on adolescent pain-related behavior via parental (Engagement) instructions.

	**Baseline predictor:**			
	**Psychologically flexible parenting**		**Parental acceptance of adolescent pain**	
**Effect (path)**	**Estimate**	**95% CI**	**Estimate**	**95% CI**
Predictor - > parental engagement instructions (a-path)	0.47	0.37 to 0.58	0.31	0.22 to 0.42

**Parental engagement instructions - > outcome (b-path)**				

**Adolescent activity-avoidance**				
*Within-dyad*	−0.03^†^	−0.06 to −0.001	−0.06^†^	−0.12 to −0.01
*Between-dyad*	0.04^†^	0.001 to 0.08	0.08^†^	0.003 to 0.15
**Adolescent activity-engagement**				
*Within-dyad*	0.04	−0.02 to 0.10	0.01	−0.01 to 0.03
*Between-dyad*	0.20	0.10 to 0.28	0.06^†^	0.03 to 0.09

**Indirect effect via parental engagement instructions (a^∗^b)**				

**Adolescent activity-avoidance**				
*Within-dyad*	−0.06^†^	−0.13 to −0.002	–0.02	−0.04 to −0.002
*Between-dyad*	0.09^†^	0.003 to 0.16	0.03^†^	0.001 to 0.05
**Adolescent activity-engagement**				
*Within-dyad*	0.02	−0.01 to 0.05	0.04	−0.02 to 0.10
*Between-dyad*	0.10	0.05 to 0.15	0.19	0.10 to 0.25
**Total effect predictor → outcome (c-path)**				
Adolescent activity-avoidance	–0.04	−0.12 to 0.05	0.01	−0.09 to 0.08
Adolescent activity-engagement	0.09^†^	0.03 to 0.16	0.17	0.09 to 0.24
**Direct effect predictor → outcome (c′-path) ^1^**				
Adolescent activity-avoidance	–0.05	−0.13 to 0.04	–0.05	−0.12 to 0.05
Adolescent activity-engagement	–0.06	−0.15 to 0.04	0.07	−0.01 to 0.14

*We refer to the template model in Figure 1 for additional help in interpreting the results presented in this table. ^1^After controlling for indirect effect. Effects indicated with ^†^ were no longer significant when applying a conservative Bonferroni correction for multiple testing.*

Finally, an indirect effect of parental psychological flexibility on higher daily adolescent activity-engagement via higher parental engagement instructions emerged, but only at the between-dyads level (*a* × *b* = 0.10, 95% CI = 0.05 to 0.15) (Table 5). This suggests that psychologically flexible parenting predicted higher overall levels of engagement instructions in parents, and that these higher overall levels of engagement instructions were associated with higher overall adolescent activity-engagement across the 14-day period. No direct effect of parental psychological flexibility on daily adolescent activity-engagement emerged when the indirect effect was controlled for (*c*′ = −0.06, 95% CI = −0.14 to 0.04) (Table 5).

#### Does Parental Acceptance of Adolescent Pain Indirectly Impact Daily Adolescent Pain-Related Behavior via Parental Engagement Instructions?

An indirect effect of parental acceptance of adolescent pain on daily adolescent activity-avoidance via daily parental engagement instructions emerged. However, the direction of this effect was opposite at the within- (*a* × *b* = −0.02, 95% CI = −0.04 to −0.002) and between-dyads levels (*a* × *b* = 0.03, 95% CI = 0.001 to 0.05) (see Table 5, right column). On the one hand, a *negative* within-dyads indirect effect emerged of parental acceptance of adolescent pain on daily adolescent activity-avoidance which was explained by daily increases in parental engagement instructions (*b_*within*_* = −0.06, 95% CI = −0.12 to −0.01). This suggests that parental acceptance of adolescent pain was predictive of higher engagement instructions in parents, and that daily increases in engagement instructions were associated with daily decreases in adolescent activity-avoidance. On the other hand, a *positive* between-dyads indirect effect emerged of parental acceptance of adolescent pain on daily adolescent activity-avoidance which was explained by lower overall levels of parental engagement instructions (*b_*between*_* = 0.08, 95% CI = 0.003 to 0.15). Parental acceptance of adolescent pain was predictive of higher overall parental engagement instructions, and these higher overall engagement instructions were associated with higher overall adolescent activity-avoidance across the 14-days. After controlling for these indirect effects, no direct effect of parental acceptance of adolescent pain on daily adolescent activity-avoidance emerged (*c*′ = 0.02, 95% CI = −0.05 to 0.09) (Table 5).

Finally, an indirect effect emerged of parental acceptance of adolescent pain on higher daily activity-engagement in adolescents via parental engagement instructions. This effect emerged at the between (*a* × *b* = 0.19; 95% CI = 0.10 to 0.25) (Table 5) but not within-dyads level. No direct effect of parental acceptance of adolescent pain on adolescent activity-engagement emerged once this indirect effect was controlled for (*c*′ = 0.001, 95% CI = −0.08 to 0.09) (Table 5).

## Discussion

Parents exert an important impact on their adolescents’ functioning in the presence of persistent pain (Palermo, 2009; Palermo et al., 2014), and in certain cases, can worsen adolescent functioning (Goubert et al., 2006; Logan et al., 2012; Hechler et al., 2015; Simons et al., 2015; Chow et al., 2016). Yet parents may also positively contribute to adaptive pain-related functioning in their child. More specifically, it has recently been argued that parental psychological flexibility may be associated with beneficial adolescent outcomes (e.g., lower disability) (Wallace et al., 2015; Timmers et al., 2019). The present study further examined whether psychologically flexible parenting and parental acceptance of adolescent pain indirectly predicted daily adolescent pain-related behavior, via their respective impact on daily parental protective responses, and/or daily instructions parents provide to their adolescent.

In line with our expectations, the findings indicated that psychologically flexible parenting and parental acceptance of adolescent pain indirectly predicted lower daily adolescent *activity-avoidance* via their impact on *lower daily parental protective* responses. Such findings are consistent with previous studies showing similar adaptive effects of parental psychological flexibility on adolescent outcomes via parental protective behavior (e.g., Timmers et al., 2019). Whereas that work was based on questionnaires administered at one moment in time, we demonstrated this indirect effect with daily data collected at multiple moments. Likewise, it was found that decreases in parental daily protective responses were associated with decreases in adolescent daily activity-avoidance within those parent-adolescent dyads where parents showed higher levels of acceptance of adolescent pain.

Furthermore, as expected, psychologically flexible parenting and parental acceptance of adolescent pain also predicted adolescent activity-avoidance via their indirect impact on *parental instructions* to engage in activities. Note, however, that these indirect effects via engagement instructions showed an opposite direction at the within-dyads versus the between-dyads level. On the one hand, we found that increased daily levels of engagement instructions within these more flexible and pain accepting parents were associated with *decreased daily* levels of activity-avoidance in their adolescents. Yet, on the other hand, we found that psychologically flexible parenting and parental acceptance of adolescent pain were also related to *higher overall* levels of adolescent activity-avoidance via their association with higher overall levels of parental engagement instructions across the 2-week period. One *post hoc* explanation for these contrasting findings is that daily increases in parental instructions to engage in more activities might momentarily lower adolescent activity-avoidance but that the persistent application of those same instructions over and over again might have the opposite effect across time. It may be that overall high levels of parental engagement instructions contribute to overall high or persistent levels of adolescents’ avoidance instead, which may adversely impact adolescent functioning on the long-term (Asmundson et al., 2012; Simons and Kaczynski, 2012; Chow et al., 2016).

In short, based on these exploratory findings, one could hypothesize that the adaptive effects of psychologically flexible parenting and parental acceptance of adolescent pain on lower levels of adolescent activity-avoidance may be explained by momentary or daily decreases in the level of protective responses and engagement instructions in these flexible or acceptant parents. Taking a step, this could suggest that these daily changes in parents’ protective responses or engagement instructions are potentially well-adapted to the daily context (e.g., how the adolescent is feeling or what activities he/she is planning on that day). This hypothesis is consistent with the idea of psychological flexibility as one’s ability to flexibly adapt behavior to the (daily) situation (Hayes et al., 2006; McCracken and Morley, 2014). Thus our findings may suggest that psychologically flexible parenting in parents of adolescents with chronic pain may be characterized by being aware of the potential consequences of being (less) protective or providing (more) engagement instructions to their adolescent.

Furthermore, this was the first study to explore the influence of psychological flexible parenting and parental acceptance of adolescent pain on adolescent *activity-engagement*. Our findings suggest that higher levels of psychologically flexible parenting indirectly contributed to higher overall activity-engagement in adolescents across the 2-week period. This indirect influence was explained by lower overall parental protectiveness on the one hand, and by higher overall engagement instructions directed at their adolescent on the other hand. Similarly, higher parental acceptance of adolescent pain indirectly influenced higher overall levels of adolescent activity-engagement across the 2-week period. However, this was only mediated by higher overall engagement instructions in parents and not by their level of protectiveness.

Finally, psychologically flexible parenting and parental acceptance of adolescent pain were only moderately related, supporting the idea that they are overlapping but unique factors (McCracken and Morley, 2014; Smith et al., 2015). We also observed little to no differences in their contribution to adolescent pain-related behavior. If anything, psychologically flexible parenting indirectly predicted adolescent activity-engagement via both protective parenting responses and engagement instructions in parents, whereas parental acceptance of adolescent pain only did so via engagement instructions.

### Future Directions and Clinical Implications

Our findings have implications for future research and clinical practice. First, they contribute to the idea that parents play a meaningful role in adolescents’ pain-related functioning, and in particular, how psychologically flexible parenting and acceptance of adolescent pain might serve an adaptive role in daily adolescent (avoidance) behavior and support the inclusion of parents in the study and treatment of adolescent pain (Palermo and Chambers, 2005; Palermo and Eccleston, 2009; Law et al., 2014).

This was also the first study to explore the effect of parental (engagement) instructions on adolescent functioning in the context of pain. That said, our initial findings on this effect do not lend themselves to a clear-cut interpretation. They suggest that instructions from parents to their adolescent that encourage them to keep doing fun or important activities when in pain may be adaptive in the short-term on a given day (i.e., associated with lower levels of avoidance). Yet high overall levels of instructions across days may be associated with high or persistent overall levels of activity-avoidance *and* activity-engagement in adolescents. These high overall levels of activity-avoidance or activity-engagement both have the potential to be maladaptive for the adolescent. For instance, persistent avoidance has been found to predict long-term negative outcomes (e.g., disability) in adolescents with chronic pain (Asmundson et al., 2012; Simons and Kaczynski, 2012). Moreover, one might also argue that high or persistent levels of engagement in activities may also predict long-term negative outcomes. Past work on adults with chronic pain demonstrated that persistent levels of engagement is associated with negative outcomes such as muscular overuse, hyperactivity, decreased well-being, and increased disability (e.g., Hasenbring and Verbunt, 2010; Crombez et al., 2012). Critically, this claim is clearly *post hoc* and awaits future replication and direct empirical testing.

Parental instructions for adolescents to engage in activities when in pain were surprisingly not associated with actual daily activity-engagement in those same adolescents. These findings therefore do not fully support the hypothesized adaptive effects of parental engagement instructions on adolescent activity-engagement. A possible explanation for these puzzling findings may be that the impact of parental instructions on their child’s behavior depends on the child’s developmental stage. For instance, it may be that these findings are specific to adolescents as our sample mainly consisted of adolescents aged between 11 and 17 years. Adolescence is a challenging period that puts pressure on the parent-adolescent relationship. It is a period in which adolescent behavioral autonomy and parental autonomy-support becomes increasingly important in fostering a healthy development of the adolescent (Baumrind, 1966; Grolnick et al., 1997; Gray and Steinberg, 1999; Joussemet et al., 2008). It may be that our findings reflect that adolescents simply do not want to follow any kind of instructions provided by their parents or even respond in the opposite way. Potentially, different effects may be found when examining the influence of parental instructions on the pain-related behavior of younger children. We believe that this is a promising area ripe to be explored. Next to examining the factors that moderate when instructions influence behavior (e.g., developmental stage) future work could also explore other types of parental instructions and their influence on adolescent behavior. Yet another interesting question would be to explore if parents’ (in)flexibility in providing instructions (e.g., adjusted to the situation or not) differently predicts adolescent pain-related functioning (also see Beeckman et al., 2019a).

Finally, upon replication, our findings may be informative for research and interventions that focus on enhancing psychological flexibility in parents to increase adolescent adaptive outcomes. Psychological flexibility is the central change process within Acceptance and Commitment Therapy (ACT), a therapy which has shown promising results for youth with chronic pain (Wicksell et al., 2005, 2007; Wicksell, 2015), and has recently been extended by incorporating parents (Kanstrup et al., 2016; Wallace et al., 2016). The current work suggests that it may be important to develop interventions directed at enhancing psychological flexibility in parents, with a specific focus on teaching parents to decrease the use of daily protective behaviors in response to adolescent pain and potentially use them in a more flexible manner. However, it was not entirely clear from our findings if these interventions should target parents’ use of instructions (to engage in activities), and how this should be done. We advocate that future work is needed that examines the effects of the (in)flexible use of protective behavioral responses and instructions in daily life on adolescent functioning before incorporating this suggestion into treatment.

### Strengths and Limitations

The present study has several strengths. It was the first to (a) investigate the indirect effects of psychologically flexible parenting and parental acceptance of adolescent pain on adolescent outcomes by using daily diary methodology, (b) introduce and examine parental daily engagement instructions as an alternative route via which these factors might have effects on adolescent functioning, and (c) examine this using multi-informant data from both parent and adolescent.

Yet, the present study also had several limitations, which may inform future research. First, no temporal associations (i.e., within-day, or day-to-day) were examined at the diary level, and as such, we cannot make interpretations of the investigated indirect effects in terms of predictability or causality. All investigated daily associations between parental responses and adolescent behavior could be interpreted in the reverse direction to that reported here. It is worth noting, however, that the proposed direction of the investigated associations stems from theory and previous empirical work (e.g., Timmers et al., 2019), lending support to the idea that psychologically flexible parenting influences adolescent outcomes via parental responses. Ideally, lagged analyses should be performed to examine such temporal associations between parental responses and subsequent changes in adolescent behavior. However, this type of analyses requires larger samples than the one described in the current study (Schultzberg and Muthén, 2018). Second, due to our limited sample size we were also not able to perform more complex analyses to directly compare the relative contribution of parental protective responses versus engagement instructions in explaining the indirect effect of psychologically flexible parenting and parental acceptance of adolescent pain on adolescent outcomes. Future research in larger samples could construct more complex mediation models with multiple mediators and predictors to examine the unique contribution of each of these factors (for an example of this analytic approach see Timmers et al., 2019). Third, although it is a strength of this study that the indirect effects were disentangled in a within- and between-dyads part, we had no pre-existing hypotheses about the effects at both levels. Our interpretations of these differences are therefore exploratory and require further investigation. This is particularly true for the finding that higher overall parental engagement instructions were associated with higher overall activity-engagement *and* activity-avoidance in adolescents. It may be that unmeasured confounding variables are responsible for this unexpected finding. Indeed, between-dyads effects are more sensitive to potential confounders than within-dyads effects, and as such, the within-dyads effects may be interpreted with more certainty (see Talloen et al., 2016). Furthermore, our *post hoc* interpretations of between-dyads effects in terms of stable (or persistent) response styles and within-effects in terms of daily variation (or flexibility) in responding in parents are preliminary. Future work should find better ways to examine (in)flexibility in parents’ responses to adolescent pain, for instance by examining statistical indicators of daily variability (for an example see Rost et al., 2016). Fourth, we used self-report measures to assess parent and adolescent behavior. Naturally these assessments are sensitive to socially desirable answering and potential memory biases. Future work could consider including observational measures to obtain a less biased, naturalistic assessment of these variables. For instance, an Electronically Activated Recorder (EAR; Mehl, 2017) may be a useful tool to assess what parents actually say to their adolescent during the day. Fifth, our sample was predominantly female (i.e., 86% girls and 93% mothers). It may be that the parent-adolescent relationships observed in this study are typical for mother-girl dyads. Future research in samples including fathers and adolescent boys with chronic pain would be useful to examine if our findings also hold for the relationship between fathers and daughters, mothers and sons, or fathers and sons. Finally, we did not correct for multiple testing. With eight models that were being tested, there was a potential risk of inflated type I errors (i.e., false positive findings). However, it was evaluated that correction of multiple testing was not appropriate for the present study because our study did not meet any of the conditions required to make such corrections (Perneger, 1998; Rothman, 2015). First, we had priori hypotheses for each of the eight models being tested. Second, we did not repeatedly test the same model in different subsamples. Finally, we favored type I errors in favor of type II errors (i.e., false negative findings). If one would have applied a conservative *post hoc* Bonferroni correction, however, and have tested regression coefficients at the 0.00625 significance level (i.e., 0.05 divided by 8 since eight different models were tested), smaller effects would have been declared non-significant (as indicated in the footnote of Tables 4, 5) but the larger effects mostly remained significant. Yet, replication of our findings by future research is warranted before strong conclusions can be made.

## Conclusion

Our findings support the claim that psychologically flexible parenting and parental pain acceptance indirectly contribute to adolescent outcomes. This was the first study to show how these parent factors predict adolescent pain-related activity-avoidance and activity-engagement on a daily basis, and suggest that this occurs indirectly via its influence on daily parental protective responses and instructions. We provided further support for the adaptive effects of psychologically flexible parenting on adolescent activity-avoidance behavior via fewer protective responses. Parents who are able to display psychological flexibility in parenting may provide their adolescent with more instructions to engage in activities (relative to instructions to avoid). Although these instructions showed short-term adaptive effects on daily adolescent avoidance behavior, our findings also suggest that parents who – on average – provide too many instructions too often might have unintended effects on adolescent behavior (i.e., high levels of activity-engagement *and* activity-avoidance). These findings contribute to our understanding of how parental psychological flexibility may impact adolescent functioning in the presence of pain.

## Data Availability Statement

The datasets generated for this study are available on request to the corresponding author.

## Ethics Statement

The studies involving human participants were reviewed and approved by the Institutional Review Boards at Boston Children’s Hospital (IRB#P0020989) and at Stanford University (IRB#39092). Written informed consent to participate in this study was provided by the participants’ legal guardian/next of kin.

## Author Contributions

MB, SH, LS, and LG made substantial contributions to the conception, design, and the acquisition and interpretation of the work. MB and TL have made substantial contributions to the data analysis.

## Conflict of Interest

The authors declare that the research was conducted in the absence of any commercial or financial relationships that could be construed as a potential conflict of interest.
